# Anxiety and depression from age 3 to 8 years in children with and without ADHD symptoms

**DOI:** 10.1038/s41598-023-42412-7

**Published:** 2023-09-16

**Authors:** Christine Baalsrud Ingeborgrud, Beate Oerbeck, Svein Friis, Pål Zeiner, Are Hugo Pripp, Heidi Aase, Guido Biele, Søren Dalsgaard, Kristin Romvig Overgaard

**Affiliations:** 1 Institute of Clinical Medicine, Division of Mental Health and Addiction, Child and Adolescent Psychiatry Unit, University of Oslo, Pb 1039 Blindern, 0315 Oslo, Norway; 2 Division of Mental Health and Addiction, Oslo University Hospital, Oslo, Norway; 3 Oslo Centre of Biostatistics and Epidemiology, Oslo University Hospital, Oslo, Norway; 4 Department of Child Health and Development, Norwegian Institute of Public Health, Oslo, Norway; 5 Institute of Clinical Medicine, University of Copenhagen, Copenhagen, Denmark; 6 Department of Child and Adolescent Psychiatry, Mental Health Services of the Capital Region, Glostrup, Denmark

**Keywords:** Risk factors, Psychiatric disorders, ADHD, Anxiety, Depression

## Abstract

Childhood anxiety and depressive symptoms may be influenced by symptoms of attention deficit/hyperactivity disorder (ADHD). We investigated whether parent- and teacher-reported anxiety, depressive and ADHD symptoms at age 3 years predicted anxiety disorders and/or depression in children with and without ADHD at age 8 years. This study is part of the longitudinal, population-based Norwegian Mother, Father and Child Cohort Study. Parents of 3-year-olds were interviewed, and preschool teachers rated symptoms of anxiety disorders, depression and ADHD. At age 8 years (n = 783), Child Symptom Inventory-4 was used to identify children who fulfilled the diagnostic criteria for anxiety disorders and/or depression (hereinafter: Anx/Dep), and ADHD. Univariable and multivariable logistic regression analyses were used. In the univariable analyses, parent-reported anxiety, depressive and ADHD symptoms, and teacher-reported anxiety symptoms at age 3 years all significantly predicted subsequent Anx/Dep. In the multivariable analyses, including co-occurring symptoms at age 3 years and ADHD at 8 years, parent-reported anxiety and depressive symptoms remained significant predictors of subsequent Anx/Dep. At age 3 years, regardless of ADHD symptoms being present, asking parents about anxiety and depressive symptoms, and teachers about anxiety symptoms, may be important to identify children at risk for school-age anxiety disorders and/or depression.

## Introduction

Excessive anxiety and depressive symptoms may appear as early as the preschool years^1^. This is also often the case for attention deficit/hyperactivity disorder (ADHD) characterized by age-inappropriate hyperactive-impulsivity and inattention^1^. Co-occurrence of anxiety and depression in children with ADHD is associated with more impairment^2–4^ and has been found to worsen the prognosis in adolescence compared to when only one of the disorders is present^5^.

A recent meta-analysis estimated the prevalence rates in young children (≤ 7 years) to be 8.5% for any anxiety disorder, 1.1% for depression and 4.3% for ADHD^6^. In children with ADHD, anxiety disorders and depression are more common than these estimates, even at preschool age. One population study of 4-year-olds reported that the respective odds for having anxiety disorders and depression were 4.5 and 17.3 times higher for children with ADHD than for children without^7^. The high co-occurrence rates between psychiatric disorders led a review 15 years ago to conclude that longitudinal, population-based studies need to document the rates of these disorders, and follow their course from preschool years onwards^8^. Yet, few preschool studies have thus far been large enough to follow through on this recommendation.

The separate courses of anxiety, depression, and ADHD have long been described in schoolchildren^9,10^. However, the few preschool studies to date have been limited by starting after the age of 4 years^11,12^, using only rating scales^13^, and/or having information from only one informant^11,14^. Following children from the age of 4 to 10 years, one population study found low to moderate stability of all three symptom clusters, with the strongest stability for ADHD^11^. We identified only one study beginning at the age of 3 years that used a diagnostic interview, examining both the course of anxiety disorders, depression, and ADHD to the ages of 6 years (n = 462)^15^, and 9 and 12 years (n = 541)^16^. The authors found that meeting the criteria for an anxiety disorder at the age of 3 was associated with significantly increased odds for later anxiety disorder at the age of 6 (odds ratio (OR) = 4.01), 9 (OR = 2.22) and 12 years (OR = 1.70), but all ORs had large confidence intervals indicating high uncertainty, with few children diagnosed with the same disorder at several time points. While the authors did not find significant stability of diagnosed depression from the age of 3 years to school age^15,16^, this was likely because of a low rate of depression at the age of 3 years (n = 6). Indeed, the analysis of symptom scores, which has higher power, revealed significant stability of anxiety, depressive and ADHD symptoms from 3 to 9 to 12 years^16^.

For more than two decades the high co-occurrence rates of anxiety and depression with ADHD, have puzzled researchers^17,18^. A recent review on anxiety and ADHD, suggested that the two have a complex relationship, in the form of one disorder leading to or affecting the course of the other^19^. That same review underlined the need to reveal the developmental nature of this comorbidity with future follow-up studies. Yet few preschool studies have followed the fluctuations between anxiety disorders, depression, and ADHD over time.

However, the abovementioned population study with enrolment at 3 years of age found that symptoms of anxiety were followed by later depressive symptoms, and vice versa^16^. Another population study found ADHD symptoms at 4 years of age to precede anxiety symptoms at school age^11^, and this finding was replicated in a recent longitudinal study (n = 258) from the age of 3 to 6 years^20^. Also, in preschool studies using diagnostic criteria, depression was found to precede anxiety disorders in 6-year-olds^15^, as well as in older schoolchildren (mean age 10.9 years)^14^. These studies were important, but where either limited by few preschoolers meeting the diagnostic criteria^15^, or by having sampled for preschoolers with depression^14^.

Treatment studies recommend that preschoolers with anxiety, depression, and/or ADHD should not be left untreated^21–23^. However, as presented above, studies on these disorders beginning in preschool age with follow-up in school age, are in demand. In fact, we found only one previous study that began at the age of 3 years and investigated the course of anxiety disorders, depression, and ADHD^15^. Also, the use of parent-report only has been cited as a limitation in other longitudinal studies of preschool anxiety disorders and ADHD^20,24^. In the present article, we extend on the above-mentioned studies by also including teacher-reported preschool psychopathology.

The aim of this prospective, longitudinal population study was to investigate whether parent- and teacher-reported anxiety, depressive and ADHD symptoms at the age of 3 years predicted anxiety disorders and/or depression in children with and without ADHD at age 8 years. We hypothesized that fulfilling diagnostic criteria for an anxiety disorder and/or depression at 8 years of age (hereinafter: Anx/Dep) would be predicted by parent-reported preschool symptoms of anxiety disorders and depression, and ADHD. Due to the known high co-occurrence rates between anxiety disorders, depression, and ADHD, we examined whether the predictive power of early anxiety and depressive symptoms to detect subsequent Anx/Dep would be altered when school-age ADHD was included in the analysis. In addition, we examined whether teacher-reported symptoms at 3 years of age contributed to the prediction of Anx/Dep at 8 years. Finally, we investigated which specific symptoms of anxiety at 3 years of age contributed to subsequent Anx/Dep.

## Methods

### Participants

The Norwegian Mother, Father and Child Study (MoBa) is an ongoing prospective population-based cohort study of Norwegian-speaking pregnant women conducted by the Norwegian Institute of Public Health, which started in 1999 (41% participation rate of invited pregnant women during the 10-year recruitment period)^25^. About 89% of the participating mothers were ethnic Norwegians, predominately white Caucasians^26^. The mean household income equaled US$ 52.200, the same as the population mean^27^. The current paper is part of a clinical sub-study described in previous publications^28,29^, oversampling children at risk for ADHD based on behaviour reported in the MoBa questionnaire administered to mothers when the children were 3 years old. In short, two groups of children were included in this study: a group with elevated scores on selected ADHD symptoms, and a random sample. Children scoring high on ADHD symptoms had scores ≥ 90^th^ percentile on an 11-item screening measure for ADHD, which included six items from the Child Behavior Checklist/1.5–5 (Can’t concentrate, Can’t sit still, Can’t stand waiting, Demands must be met immediately, Gets into everything, Quickly shifts activities)^30^, and five items from the DSM-IV-TR (Easily distracted, Difficulty waiting his/her turn, Difficulty sustaining attention, Talks excessively, Does not seem to listen)^31^ (n = 2,798). They were eligible for participation along with randomly selected children (n = 654). About 35% of the invited families participated in the initial assessment, and from 2007 to 2011, 1,195 children (mean age 3.5 years; age range 3.1–3.8 years) took part in a one-day clinical assessment including diagnostic interviews with parents using the Preschool Age Psychiatric Assessment (PAPA)^32^. With very few exceptions, the parent participating was the mother. The teachers’ screening questionnaires were sent by mail to the parents who gave them to the teachers. About 93% of the invited teachers mailed their completed questionnaires back to the study administrator. A small number withdrew their consent to participate during the study, leaving 1,180 participants (618 boys) at the 8-year follow-up. A total of 67% (n = 783; 415 boys) of the mothers who participated when their child was 3 years old also completed the questionnaires when their child was 8 years old. Among the 783 children with parent-report at age 3 and 8 years, 393 also had information from teachers.

### Measures

#### Measures at 3 years of age

Psychiatric assessment of the children was based on PAPA interviews with parents. The PAPA is a semi-structured interview developed for children from 2 to 5 years. With the PAPA, the interviewer asks questions until they can decide whether the symptoms described meet the definitions provided in a glossary. In a study of test–retest reliability of the PAPA interview, the test–retest intraclass correlations (ICCs) for symptoms of ADHD, any anxiety disorder and depression were 0.80, 0.74 and 0.71, respectively^33^. In the ADHD substudy the ICCs were previously found to be acceptable (> 0.86)^28,29^. The interviews were conducted by trained graduate psychology students blinded to the questionnaire ratings, under the supervision of a specialist in child psychology or psychiatry. Only a subsample of teachers (about 56%) received the Norwegian version of the Early Child Inventory-4 (ECI-4)^34^ containing 108 items corresponding to the symptom lists of the DSM-IV child psychiatric disorders.

#### Predictors

##### Parents’ classifications

Based on information from the PAPA interview, symptoms of anxiety, depression and ADHD were counted as “present” or “not present” during the previous three months, and then computed into symptom sum scores. In line with the PAPA guidelines, the anxiety sum score included seven symptoms of specific phobia, three symptoms of social phobia, seven symptoms of separation anxiety and six symptoms of generalized anxiety. Following the algorithm for general anxiety, these symptoms were only counted when the criterion “worries” was reported. The depression sum score consisted of nine symptoms (depressed mood, tearfulness, easily frustrated, irritability, anhedonia, anergia, indecisiveness, feels unloved and suicidal thoughts). The ADHD sum score consisted of 18 symptoms (nine of hyperactivity/impulsivity, and nine of inattention).

##### Teachers’ ECI-4 classifications

The ECI-4 anxiety, depressive and ADHD symptoms were rated on a four-point Likert scale (never, sometimes, often, very often; range 0–3), and then dichotomized to not present (0, 1 = 0) and present (2, 3 = 1) in line with the manual, and computed to symptom count scores. The anxiety sum score included one symptom of specific phobia, two symptoms of social phobia, one symptom of separation anxiety and seven symptoms of general anxiety. For generalized anxiety, symptoms were only counted when the items “worries that other children can do better than he/she can” or “worries more than other children” were first endorsed. The depression sum score consisted of 10 symptoms; symptoms were only counted when at least one of the items “depressed”, “irritable mood”, “sensitive or tearful” or “shows little interest in fun activities or playing with other children” was endorsed. The ADHD sum score consisted of nine items from the hyperactivity/impulsivity subscale and nine items from the inattention subscale, all counted as not present or present.

##### Background variables

Birth date and child sex were obtained from the Norwegian Medical Birth Registry. Length of parental education was obtained from the first MoBa assessment during pregnancy and reported in mean number of years.

All correlations between measures at the age of 3 years (symptom sum scores from parents’ and teachers’ classifications and background variables) were low to moderate (Pearson’s r range (–0.19 to 0.36)).

#### Measures at age 8 years

The parents completed the Child Symptom Inventory-4 (CSI-4), a questionnaire with items and algorithms for diagnoses, derived from the DSM-IV diagnostic criteria^35^. The CSI-4 has been found to be reliable and valid, with a temporal stability over a four-year period for most symptom categories^36^. In line with the manual, the CSI-4 was rated on a four-point Likert scale (never, sometimes, often, very often; range 0–3)^35^, and was dichotomized to being not present (0, 1 = 0) or present (2, 3 = 1). In line with the algorithms in the CSI- manual, the diagnostic cut-off scores were set to the minimum number of symptoms necessary for the DSM-IV diagnoses of anxiety disorders, depression, and ADHD, respectively. Cronbach’s alphas for the CSI-categories were acceptable (ranged from 0.71—0.80 for the anxiety disorders and depression and was 0.88 for ADHD).

##### Outcome

Anxiety disorders included social phobia, separation anxiety and general anxiety disorder. For social phobia, the CSI-4 lists four symptoms, at least three of which had to be endorsed to fulfil the diagnostic criteria. For separation anxiety, the CSI-4 lists eight symptoms, at least three of which had to be endorsed. For generalized anxiety disorder, eight items are listed; where the modified criteria “Is overconcerned about abilities in academic, athletic or social activities” or “Has difficulty controlling worries” had to be endorsed for the other symptoms to be counted. For generalized anxiety disorder to be present, at least three symptoms had to be endorsed. Depression included major depression and dysthymia; CSI-4 lists 10 symptoms for major depression and eight for dysthymic disorder, with six symptoms overlapping the two disorders. Major depression was defined as five or more symptoms endorsed, at least one of which is “depressed mood” or “irritable mood”, or “loss of interest or pleasure”. To meet the criteria for dysthymia, “Depressed for most of the day” and “Irritable most of the day” had to be endorsed, in addition to two other relevant symptoms. We did not include specific phobias, as the CSI-4 does not count the number of phobias and having more than one specific phobia has been shown to be important for increased impairment and poor prognosis^37^. We found specific phobias to be frequent at 8 years of age (n = 231), and 46 of these children also met the criteria of another diagnosis of anxiety or depression.

Only five children fulfilled the criteria for depression without also meeting the criteria for at least one anxiety disorder. Mean number of anxiety symptoms at 3 years of age were similar in 8-year-olds with anxiety only, with anxiety and co-occurrent depression, or with depression only. We therefore merged these three groups into an Anx/Dep group that consisted of 8-year-old children with at least one anxiety disorder and/or depression classified in accordance with the CSI-4.

For ADHD, the CSI lists nine symptoms of inattention (IA) and nine symptoms of hyperactivity-impulsivity (HI), and at least 6 symptoms of IA and/or HI had to be endorsed.

A total of 783 (66%) children participated in the assessment at 8 years of age. We checked for differences in symptoms at 3 years of age between responders and non-responders; there were no clear group differences in mean number of symptoms of anxiety, depression and ADHD reported by parents and teachers at 3 years. The distribution of girls and boys was similar in the two groups. The parents of the responders were slightly better educated in number of years (mean = 14.94, SD = 2.21) than the parents of non-responders (mean = 14.64, SD = 2.35, *p* = 0.04).

## Ethics

Parents who agreed to participate in MoBa and the ADHD study signed an informed consent form. MoBa and the initial data collection were based on a license from the Norwegian Data Protection Agency and was approved by the Regional Committees for Medical and Health Research Ethics. The MoBa cohort is currently regulated by the Norwegian Health Registry Act. The current study was approved by the Regional Committees for Medical and Health Research Ethics (2017/1276) and was performed in accordance with guidelines and regulations as stated in the Declaration of Helsinki.

## Statistical analysis

Statistical analyses were performed using SPSS Statistics for Windows, Version 26, and internal validation was conducted using R with the R package rms^38^. Mann–Whitney U tests were used to analyse differences between scores of continuous variables, and Pearson chi-square tests were used to compare categorical variables. In univariable logistic regression models, we estimated the predictors’ contributions to Anx/Dep at 8 years of age. First, we made a multivariable logistic regression model including children with complete datasets (n = 760) with backward stepwise elimination, where those predictors that contributed to the model at *p* < 0.2 were kept in the analysis. We added interaction terms of age 3-year symptoms of anxiety and ADHD, and depression and ADHD, respectively, to the model. Second, we added ADHD (categorical variable) at 8 years to the multivariable model. Finally, we used a logistic regression model to test symptoms of the specific anxiety disorders as predictors of Anx/Dep at 8 years, and again ADHD at 8 years was added to the model. In all multivariable models, parental education reported in pregnancy was included. ORs and 95% confidence intervals (CIs) were computed. Tests were two-tailed, and significance levels was set at 0.05.

The multivariable logistic regression model with backward stepwise elimination was internally validated by bootstrapping using 1,000 bootstrap samples to assess overfitting (i.e. better model performance in the study sample than in new samples with other subjects)^39^. The “apparent” predictive accuracies are usually overestimated in multivariable logistic regression models, and internal validation corrects for this bias (“optimism”). Apparent (estimated directly from the dataset that was used to develop prediction model) and optimism-corrected (subtracting average optimism from apparent performance) area under the curves (AUC/C-statistics) were computed, ranging from 0.5 to 1.0, with larger values corresponding to superior accuracies. Also, we assessed the model performance in the statistical terms of the R-squared (R^2^) and calibration slope (where 1.00 is ideal and estimates below 1.00 indicate overfitting).

## Results

At 8 years of age, 7.8% (61/783) of the children were in the Anx/Dep group (Table 1). Of these, 44% (27/61) were also classified with ADHD. There were similar rates of the different anxiety disorders and depression at 8 years in boys and girls, but more boys than girls were classified with ADHD (13.3% vs. 8.1%, χ^2^ = 5.47,* p* = 0.019).

**Table 1:** Parent-reported child diagnoses at 8 years of age (n = 783).

	n	%	n boys/girls	% boys/girls
Diagnoses ª				
Any anxiety disorder	56	7.2	29/27	7.0/7.3
Social phobia	15	1.9	7/8	1.7/2.2
Separation anxiety	22	2.8	12/10	2.9/2.7
Generalized anxiety disorder	33	4.2	20/13	4.8/3.5
Depression	18	2.3	12/6	2.9/1.6
Any anxiety disorder or depression (Anx/Dep)	61	7.8	33/28	8.0/7.6
More than one anxiety disorder or depression	19	2.4	11/8	2.7/2.2
ADHD	85	10.9	55/30	13.3/8.1*
Any anxiety disorder or depression, and ADHD	27	3.4	13/14	3.1/3.8

ª All diagnoses were classified by the Child Symptom Inventory-4; * *p* = 0.019; ADHD, attention-deficit/hyperactivity disorder.

At 3 years of age, there were significantly higher parent-reported symptom scores of anxiety disorders, depression and ADHD among the children in the Anx/Dep group compared with children without any anxiety disorder or depression (Table 2).

**Table 2:** Descriptives and group comparisons.

Predictor variables at 3 years	All children		No anxiety disorder or depression 8 years		Any anxiety disorder or depression 8 years (Anx/Dep)			
Parent-reported symptoms	n	M (SD)	n	M (SD)	n	M (SD)	Z	*p*
Total anxiety	783	1.08 (1.65)	722	0.97 (1.47)	61	2.37 (2.79)	–4.11	< 0.001
Specific phobia	783	0.29 (0.66)	722	0.26 (0.63)	61	0.59 (0.92)	–3.52	< 0.001
Social phobia	783	0.10 (0.45)	722	0.08 (0.37)	61	0.39 (0.90)	–4.24	< 0.001
Separation anxiety	783	0.48 (0.89)	722	0.44 (0.85)	61	0.98 (1.22)	–4.42	< 0.001
Generalized anxiety	783	0.21 (0.60)	722	0.20 (0.58)	61	0.41 (0.82)	–2.70	0.007
Depression	781	0.32 (0.64)	720	0.29 (0.60)	61	0.76 (0.91)	–5.02	< 0.001
ADHD	783	3.97 (3.84)	722	3.77 (3.73)	61	6.26 (4.38)	–4.62	< 0.001
Teacher-reported symptoms								
Total anxiety	393	0.14 (0.44)	365	0.12 (0.36)	28	0.33 (1.03)	–0.60	0.546
Depression	393	0.12 (0.49)	365	0.13 (0.50)	28	0.04 (0.19)	–0.90	0.370
ADHD	393	1.61 (3.04)	365	1.59 (3.02)	28	1.86 (3.32)	–0.68	0.495
Covariates								
Parental education (years)	763	14.94 (2.21)	704	15.07 (2.16)	59	13.43 (2.24)	–5.00	< 0.001
		% (n)		% (n)		% (n)	χ	
Sex (boys)	783	52.7 (413)	722	52.6 (380)	61	54.1 (33)	0.05	0.826
ADHD 8 years (yes)	783	10.9 (85)	722	8.0 (58)	61	44.3 (27)	76.29	< 0.001

M, mean; SD, standard deviation; ADHD, attention-deficit/hyperactivity disorder; Symptom sum scores at 3 years were computed from the Preschool Age Psychiatric Assessment (parents) and the Early Child Inventory-4 (teachers); Diagnoses at 8 years were classified by the Child Symptom Inventory-4.

In the univariable regression analyses, all the parent-reported predictors at 3 years (symptoms of anxiety disorders, depression and ADHD), and the teacher-reported anxiety symptoms, contributed significantly to Anx/Dep, but sex did not (Table 3). In the multivariable analyses, parent-reported symptoms of anxiety disorders and depression remained significant predictors of Anx/Dep, whereas ADHD symptoms contributed only slightly to this model (OR = 1.07, *p* = 0.053).

**Table 3:** Logistic regression analyses with symptom counts at 3 years predicting diagnoses of anxiety disorder and/or depression (Anx/Dep) at 8 years, with internal validation of the models.

Predictors	Univariable analyses				Multivariable model^a, b^				Multivariable model including ADHD at 8 years^a, b^			
	B (SE)	OR	95% CI	*p*	B (SE)	OR	95% CI	*p*	B (SE)	OR	95% CI	*p*
Parent-reported symptoms												
Anxiety	0.38 (0.06)	1.38	1.24–1.56	< 0.001	0.26 (0.06)	1.29	1.14–1.47	< 0.001	0.26 (0.07)	1.30	1.14–1.48	< 0.001
Depression	0.78 (0.15)	2.18	1.61–2.95	< 0.001	0.56 (0.17)	1.75	1.25–2.45	0.001	0.59 (0.18)	1.80	1.27–2.54	0.001
ADHD	0.14 (0.30)	1.15	1.09–1.22	< 0.001	0.07 (0.03)	1.07	1.00–1.14	0.053				
Teacher-reported symptoms												
Anxiety^c^	0.61 (0.30)	1.84	1.03–3.28	0.040								
Depression^c^	–0.79 (0.88)	0.45	0.08–2.56	0.370								
ADHD^c^	0.03 (0.06)	1.03	0.91–1.15	0.658								
Sex	0.06 (0.27)	1.06	0.63–1.79	0.826								
Constant					0.69 (0.98)	1.99		0.482	0.08 (1.02)	1.09		0.934
	Apparent	Optimism corrected	Apparent	Optimism corrected								
AUC/C-statistics	0.78	0.76	0.82	0.81								
R^2^	0.21	0.18	0.28	0.25								
Slope	1	0.93	1	0.93								

^a^ The multivariable models were adjusted for parental education length; ^b^ Included in the analysis were children with complete datasets (n = 760) ^c^ Subsample with teachers’ report, n = 393; ADHD, attention-deficit/hyperactivity disorder; AUC, Area Under the Curve; OR, odds ratio; CI, confidence interval; Symptom sum scores at 3 years were computed from the Preschool Age Psychiatric Assessment (parents) and the Early Child Inventory-4 (teachers); Diagnoses at 8 years were classified by the Child Symptom Inventory-4.

In the multivariable model from the teacher subsample, the teacher-reported symptoms of anxiety disorders or depression at 3 years did not contribute significantly to Anx/Dep.

There were no significant effect modifications of the multivariable model when adding interaction terms of age 3 symptoms of anxiety disorders and ADHD, or age 3 symptoms of depression and ADHD, respectively (statistics not shown).

When entering ADHD at 8 years in the final multivariable regression model, parent-reported anxiety and depressive symptoms at 3 years remained significant predictors of Anx/Dep (ORs = 1.30 and 1.80, respectively).

Apparent and optimism-corrected AUC/C-statistics, R^2^ and calibration slope for the multivariable models are shown in Table 3. The optimism-corrected C-statistics (0.78–0.81) indicate that the models should be considered reasonable to strong.

Finally, we checked which symptoms of the specific anxiety disorders at age 3 contributed to Anx/Dep; only symptoms of social phobia remained a significant predictor in the multivariable model, and this remained after including ADHD at 8 years (Table 4).

**Table 4:** Logistic regression analyses with parent-reported symptom counts of specific anxiety disorders, depression and ADHD at 3 years predicting diagnoses of anxiety and/or depression (Anx/Dep) at 8 years.

Predictors	Univariable analyses				Multivariable model^a, b^				Multivariable model including ADHD at 8 years ^a, b^			
	B (SE)	OR	95% CI	*p*	B (SE)	OR	95% CI	*p*	B (SE)	OR	95% CI	*p*
Parent-reported symptoms												
Specific phobia	0.53 (0.15)	1.70	1.27–2.26	< 0.001	0.28 (0.18)	1.33	0.94–1.87	0.107	0.27 (0.18)	1.31	0.92–1.88	0.137
Social phobia	0.84 (0.18)	2.31	1.62–3.30	< 0.001	0.83 (0.21)	2.28	1.50–3.46	< 0.001	0.92 (0.22)	2.51	1.63–3.86	< 0.001
Separation anxiety	0.46 (0.11)	1.59	1.28–1.97	< 0.001								
Generalized anxiety	0.41 (0.16)	1.51	1.10–2.08	0.011								
Depression	0.78 (0.15)	2.18	1.61–2.95	< 0.001	0.63 (0.17)	1.87	1.34–2.62	< 0.001	0.68 (0.18)	1.96	1.38–2.89	< 0.001
ADHD	0.14 (0.30)	1.15	1.09–1.22	< 0.001	0.08 (0.04)	1.09	1.02–1.17	0.015				
Constant					0.98 (1.00)	2.65		0.328	0.46 (1.03)	1.58		0.659

^a^ The multivariable models were adjusted for parental education length; ^b^ Included in the analysis were children with complete datasets (n = 760); ADHD, attention-deficit/hyperactivity disorder; OR, odds ratio; CI, confidence interval; Symptom sum scores at 3 years were computed from the Preschool Age Psychiatric Assessment; Diagnoses at 8 years were classified by the Child Symptom Inventory-4.

## Discussion

Parent-reported anxiety and depressive symptoms when children were 3 years old predicted the fulfilment of diagnostic criteria for anxiety disorder and/or depression (i.e., Anx/Dep) at 8 years of age. For each one-unit increase in the symptom sum score of anxiety and depression at 3 years, the estimated odds for Anx/Dep increased by 38% and 78%, respectively. Anxiety and depressive symptoms remained significant predictors of subsequent Anx/Dep in the multivariable analysis, even after including co-occurring ADHD at 8 years in the model. Parent-reported ADHD symptoms at 3 years was a significant predictor of Anx/Dep in the univariable analysis, though the estimate was attenuated when adjusted for the effects of anxiety and depressive symptoms at 3 years. Teacher-reported anxiety symptoms at 3 years also predicted Anx/Dep but did not contribute significantly when including the parent-reported symptoms. Symptoms of all the specific anxiety disorders at 3 years predicted Anx/Dep at 8 years in the univariable analysis. Among these, only symptoms of social phobia contributed to Anx/Dep when controlling for symptoms of other anxiety disorders as well as depression and ADHD at 3 years.

Consistent with our hypothesis and in line with previous research^14,16^, parent-reported symptoms of anxiety disorders and depression at 3 years significantly contributed to Anx/Dep at 8 years. This was in line with the results of another population study that analysed continuous symptom scores from age 3 to 9 and 12 years^16^. However, that same study, when using diagnostic categories, did not find that depression predicted subsequent anxiety disorders or depression. Together with our findings, this lends support to a previous review suggesting that preschool anxiety and depressive symptoms below diagnostic threshold may be clinically relevant; by excluding them, there is a risk of overlooking young children at risk for these disorders^40^.

In line with our hypothesis, we found that parent-reported ADHD symptoms at 3 years significantly predicted Anx/Dep at 8 years but contributed only slightly to the multivariable analysis. Still, clinicians should be aware that preschool ADHD symptoms increase the risk for anxiety and depression years later. In another population study, without oversampling for ADHD symptoms and with continuous interview symptom scores at both time points, ADHD symptoms at 3 years were not found to be associated with depressive symptoms at 9 years, and only slightly increased the risk for subsequent anxiety symptoms (incidence risk ratio = 1.05)^16^. A recent study found ADHD symptoms in 3-year-olds to predict higher levels of anxiety symptoms later on^20^; however, again unlike our study, they used continuous symptom scores at both time points, and sampled participants with more severe behavioural problems (aggression) than in the present study. Also, we previously reported moderate diagnostic stability of ADHD from 3 to 5 and 8 years^41,42^ suggesting that stability of ADHD symptoms across time in our population-based sample may be too low to detect a strong association between ADHD and subsequent Anx/Dep.

Nearly one-third of children who met the criteria for ADHD at 8 years also met the criteria for at least one co-occurring anxiety disorder or depression at the same time point. This is in line with previous work documenting high comorbidity rates among these disorders^43,44^. Given the high co-occurrence rates, we included ADHD at 8 years in the model, as the course of anxiety and depression could be different for children with ADHD. However, adding ADHD at 8 years did not substantially change the odds ratios for preschool anxiety and depressive symptoms predicting subsequent Anx/Dep. Our results indicate that these conditions may have separate trajectories throughout preschool years, consistent with findings in older children^45^. This underlines the need for clinicians to assess each disorder independently even in young children.

In the present study, we extended on previous longitudinal preschool studies by including teacher reports. The correlation between parent-reported and teacher-reported anxiety symptoms at 3 years was low (0.15), in line with a previous finding^46^. Yet, teacher-reported anxiety symptoms, but not depressive or ADHD symptoms, predicted subsequent Anx/Dep, suggesting that asking preschool teachers about anxiety symptoms may be useful in identifying young children at risk. Still, in the multivariable models, the teacher-reported anxiety symptoms at 3 years did not contribute to Anx/Dep when the parent-reported symptoms were included. In line with this finding, the inclusion of multiple sources in studies examining comorbidity has previously been found to be challenging because of significant disagreement between parent and teacher informants^47^. Both bias in the informants’ ratings and differences in the children’s symptoms in various settings may contribute to discrepancies in the reports^48^. Even for school children, previous studies have concluded that children’s problems with anxiety and depression often remain largely hidden to teachers^49,50^. Therefore, the uncertainty in the teachers’ ratings in our study is expected, as differentiating between normal behaviour and symptoms in children as young as 3 years may be even more difficult than for older children^8^. Taken together, these findings indicate that clinicians may prioritize parent-report when assessing preschoolers for anxiety and depression.

Among the symptoms of the separate anxiety disorders at 3 years, only symptoms of social phobia predicted Anx/Dep in a multivariable logistic regression model including depression and ADHD symptoms. Symptoms of social phobia are related to the temperamental trait behavioural inhibition (BI), which is characterized by reticence in new situations with unfamiliar people and objects^51^. Prospective studies of BI in early childhood found that this temperamental trait most strongly predicts subsequent social anxiety^52,53^, but also predicts other anxiety disorders^54^.

## Strengths and limitations

Strengths of the current study include the population-based cohort design, prospective follow-up, use of parent diagnostic interviews, and inclusion of teacher reports at 3 years of age. The study also had several limitations. It is worth noting that this study was designed to estimate risk from preschool symptoms, and not to detect causal effects on anxiety and depression. Selection bias due to attrition has been previously described^28,29^. We found no differences between responders and non-responders in sex distribution or mean number of symptoms reported by parents and teachers at 3 years, and only slight differences in mean parent education, indicating that attrition at 8 years should not have biased the results considerably. Teacher reports were only available for a subsample of the 3-year-olds, reducing statistical power, yet the power was sufficient to detect that teacher-reported anxiety symptoms at 3 years contributed significantly to subsequent anxiety disorders and depression in the univariable analysis. We used continuous symptom counts, reporting ORs for each additional symptom at 3 years on Anx/Dep and not the absolute risks. However, few children generally met the diagnostic criteria for anxiety disorders and depression at 3 years. At age 8 years, we only had parent questionnaire ratings of symptoms, not equivalent to diagnoses which would entail a broader assessment. Children’s symptoms of Anx/Dep were not reported by teachers at 8 years, adding to the risk of shared method variance and reporter bias (e.g., anxious or depressed mothers may report more symptoms in their children). Information from parents is however the most common measure of anxiety and depression in schoolchildren, as teachers frequently overlook children with these symptoms, perhaps because these children are often well behaved^50^. The outcome included both anxiety disorders and depression, and the two groups were not sufficiently large to divide them. Furthermore, as presented in Table 1, only 5 of the 18 children with depression did not meet the criteria for a co-occurrent anxiety disorder. We did not include specific phobia in the outcome because using the algorithms in the CSI manual gave a high occurrence rate for specific phobia at 8 years (29.5%) but did not measure the number of specific phobias, and having more than one specific phobia has been shown to be important^37^. The children with specific phobia had significantly lower overall anxiety and depressive symptom scores at 3 years than the children who had other anxiety disorders at 8 years. The participants in this study were oversampled for elevated symptoms of ADHD at 3 years; this might have excluded quiet or introverted preschoolers who were perhaps prone to anxiety or depression. Yet, investigating the course of anxiety and depression in preschoolers with high activity levels is important, given the high co-occurrence rates of anxiety disorders and ADHD, and depression and ADHD, throughout childhood. Finally, although there was a risk of overfitting logistic regression models to the present sample, the optimism-corrected C-statistics (0.78–0.81) suggest that the models should be considered reasonable to strong.

## Conclusions and future directions

The findings in this study suggest that to detect preschoolers at risk for school-age anxiety disorders or depression, clinicians should ask parents about anxiety and depressive symptoms and ask teachers about anxiety symptoms, both in children with and without co-occurrent ADHD symptoms. They should be aware that anxiety and depressive symptoms are easily overlooked in young children. Also, the complexity increases when these disorders co-occur with ADHD. Therefore, the use of standardized measures (such as questionnaires or diagnostic interviews) is recommended. The results from these assessments will be central when tailoring individual treatments whether it is ADHD-focused parent-training programs in line with the Nice ADHD guidelines^55^ or age-adapted forms of cognitive behavioral therapy for anxiety and depression^21^. Also, prevention efforts, such as parent intervention programmes^56^, have been promising in preschoolers with symptoms of anxiety disorders. Future studies should aim to identify shared risk factors, contributing to the high co-occurrence rates for anxiety disorders, depression, and ADHD. As not all preschoolers with anxiety or depressive symptoms develop subsequent disorders, it will be important to identify moderating factors on the course of these symptoms to school age.

## Data Availability

The data that support the findings of this study are available from the corresponding author, upon reasonable request.
